# Practical Recommendations for the Use of the GeneSwitch Gal4 System to Knock-Down Genes in *Drosophila melanogaster*

**DOI:** 10.1371/journal.pone.0161817

**Published:** 2016-08-29

**Authors:** Filippo Scialo, Ashwin Sriram, Rhoda Stefanatos, Alberto Sanz

**Affiliations:** Institute for Cell and Molecular Biosciences, Campus for Ageing and Vitality, University of Newcastle, Newcastle-upon-Tyne, NE4 5PL, United Kingdom; CNRS UMR7622 & University Paris 6 Pierre-et-Marie-Curie, FRANCE

## Abstract

*Drosophila melanogaster* is a popular research model organism thanks to its’ powerful genetic tools that allow spatial and temporal control of gene expression. The inducible GeneSwitch Gal4 system (GS) system is a modified version of the classic UAS/GAL4 system which allows inducible regulation of gene expression and eliminates background effects. It is widely acknowledged that the GS system is leaky, with low level expression of UAS transgenes in absence of the inducer RU-486 (the progesterone analog that activates the modified GAL4 protein). However, in the course of our experiments, we have observed that the extent of this leak depends on the nature of the transgene being expressed. In the absence of RU-486, when strong drivers are used to express protein coding transgenes, leaky expression is low or negligible, however expression of RNA interference (RNAi) transgenes results in complete depletion of protein levels. The majority of published studies, using the GS system and RNAi transgenes validate knock-down efficiency by comparing target gene mRNA levels between induced and non-induced groups. Here, we demonstrate that this approach is lacking and that both additional control groups and further validation is required at the protein level. Unfortunately, this experimental limitation of the GS system eliminates “the background advantage”, but does offer the possibility of performing more complex experiments (e.g. studying depletion and overexpression of different proteins in the same genetic background). The limitations and new possible applications of the GS system are discussed in detail.

## Introduction

**Fig 1:**
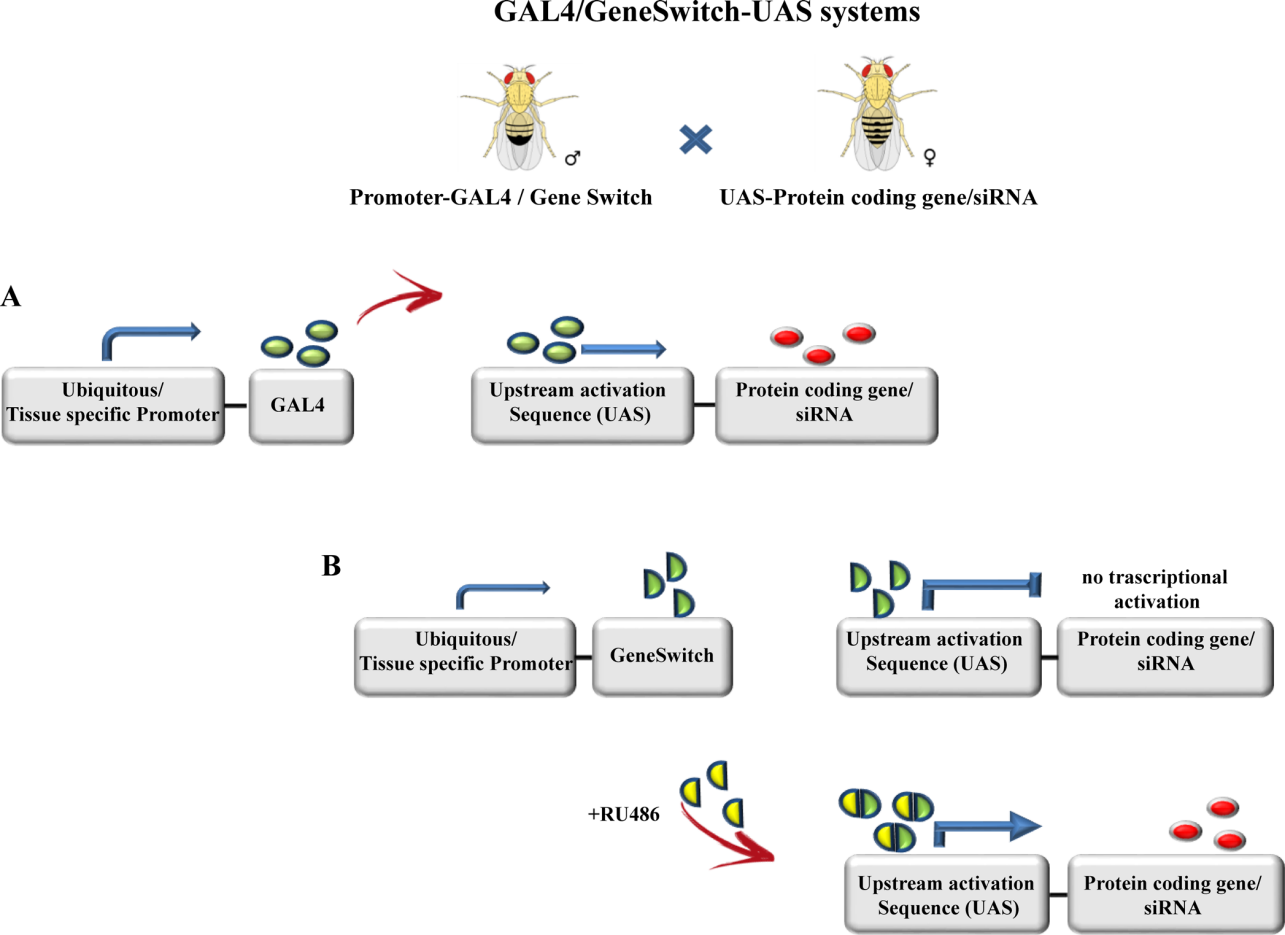
Schematic representation of the GAL4/GeneSwitchGal4-UAS systems. (A) The GAL4-UAS system allows spatial control of gene expression. (B) The GeneSwitch (GS) system allows temporal control of gene expression thanks to a modified GAL4 protein that is active only when the synthetic progesterone analogue (mifespristone, RU-486) binds to the fused progesterone steroid receptor. In absence of RU-486, GAL4 activity is maintained at a minimum. Pictures of *Drosophila* male and female where obtained from: https://commons.wikimedia.org/wiki/File:Biology_Illustration_Animals_Insects_Drosophila_melanogaster.svg.

*Drosophila melanogaster*, popularly known as the fruit fly, is a powerful model organism to study genetic interactions, including those associated with human disease [1]. An extensive collection of genetic tools developed by the fly community over the last 50 years support the activity of researchers working with *Drosophila*. A non-exhaustive list of those tools includes [2–6]: (i) P-element mediated mutagenesis, (ii) site-specific recombination through the FLP-FRT system, (iii) site-specific transgene insertion using the PhiC31 integrase, (iv) silencing of specific genes using RNA interference (RNAi) and (v) genome editing using CRISPR-Cas9. The majority of the aforementioned techniques are in some way coupled to the GAL4-UAS (upstream activator sequence) binary expression system that allows spatial and temporal regulation of gene expression. The GAL4-UAS system, developed by Brand and Perrimon to study development [6] has two components: (i) the yeast transcription factor GAL4 that is placed under the control of either a ubiquitous or tissue-specific promoter, and (ii) a gene of interest cloned downstream of a UAS sequence. The Transgene is expressed upon binding of GAL4 to the UAS sequence (Fig 1A).

Spatial control of gene expression is simple using GAL4-UAS and relies on the use of tissue-specific promoters. However, temporal control of gene expression requires co-expression of GAL4 with a temperature sensitive repressor GAL80^ts^ [7]. GAL80^ts^, however, has two different caveats. Firstly, its use requires that rearing temperature is tightly controlled and secondly, full transgene expression can only be attained at high temperatures (≥29°C). Such conditions are not always suitable, in particular when studying genes whose miss-expression is lethal during development. In order to overcome these problems, the chemically inducible GeneSwitch-GAL4 (GS) system was developed[8]. GS uses a modified GAL4 protein fused to a progesterone steroid receptor, allowing the regulation of its GAL4 activity via the presence or absence of the synthetic progesterone analogue mifespristone (RU-486). In the presence of RU-486, the transactivating activity of GAL4 is enhanced leading to increased transgene expression. Conversely, in the absence of RU-486 GAL4 activity is maintained at a minimum (Fig 1B). Since the modified GAL4 protein is active in the absence of RU-486, appropriate controls must be used to quantify the extent of the leak in different experimental conditions. Indeed, expression of reporter proteins such as lacZ or GFP has been previously shown in non-induced conditions [9], however the differences between induced and non-induced conditions were clear and so considered acceptable.

However, we have observed that the level of transgene expression in non-induced conditions is dependent on the nature of the transgene. For example, we observed negligible or minimal expression of protein coding transgenes in the absence of RU-486, using two different promoters: (i) *daughterless-Gene-Switch* (*daGS)* and (ii) *tubulin-Gene-Switch* (*tubGS)*. On the other hand using the same drivers to express RNA interference (RNAi) transgenes to knock-down gene expression resulted in a significant decrease in target gene protein levels which was independent of the presence of RU-486. In fact, when using a strong driver such as *tubGS* no significant differences in gene expression were observed between induced and non-induced flies. Importantly, we show that validation of target knock-down by comparing mRNA levels of induced versus non-induced flies can be misleading and that inclusion of controls which do not carry the RNAi transgene as well as further validation at the protein level is advisable. Finally, we discuss based on the observations we report here, which experiments should and should not be performed using the GS system.

## Material and Methods

### Fly husbandry

Virgin females carrying a *daughterless (daGS)*- or *tubulin*-*GeneSwitch* (*tubGS)* driver were crossed with (i) the following RNAi lines: 13131 (*CG6020*), 46799 (*CG3683*) 42162 (*CG8905*), 40466 (*CG3731*), 30892 (*CG11015*), 34664 (*CG3612*), (ii) UAS-lines carrying protein coding transgenes (NDI1, AOX, LacZ and ND-42-HA) or (iii) a wild type stock of Dahomey males [10]. Additionally, Dahomey virgin females were crossed with males carrying RNAi transgene against *CG6020* as described above. Flies were collected following eclosion and transferred to new food for mating for 24 hours before being sorted for experiments. Mated 5 day old female flies maintained at 25°C were used for all experiments. Flies were maintained on standard media (1% agar, 1.5% sucrose, 3% glucose, 3.5% dried yeast, 1.5% maize, 1% wheat, 1% soya, 3% treacle, 0.5% propionic acid, 0.1% Nipagin) with a controlled 12hr:12hr light:dark cycle. All RNAi lines were obtained from the Vienna Drosophila Resource Center (VDRC) [5]. UAS-*NDI1* and *UAS*-AOX have been previously described [11]. *UAS-ND42-HA* flies were a kind gift from the laboratory of Prof Hugo Bellen [12], *UAS-LacZ* was obtained from the Bloomington Drosophila Stock Center (BDSC) [13]. The *daGS* and *tubGS* drivers were a generous gift from the laboratories of Dr Veronique Monnier and Dr Scott Pletcher respectively.

### qPCR

RNA extraction, cDNA synthesis and qPCR were performed as described in [14]. Primer sequences are available upon request.

### Western blots

Sample preparation and western blotting were performed as described in [15]. The primary antibodies, employed together with the appropriate secondary antibodies, were as follows: anti-AOX described in[16], used at 1:100,000; anti-NDI1 described in [10], used at 10,000; anti-LacZ (Abcam, Oregon, USA), used at 1:1,000; anti- SOD2 (Abcam, Oregon, USA), used at 1:1,000; anti-NDUFA8 and anti-NDUFA9 (a gift from Prof Howy Jacobs (University of Helsinki)), used at 1:1,000 and 1:2,500 respectively; anti-ATP5A (Abcam, Oregon, USA), 1:500,000; anti-HA (Human influenza hemagglutinin), used at 1:1,100; anti-beta Tubulin (Abcam, Oregon, USA), used at 1:2,000 and anti-GAPDH (Everest Biotech, Oxfordshire, United Kingdom), 1:40,000. The secondary antibodies were as follows: HRP-conjugated horse anti-mouse IgG [H+L] (Vector Laboratories, Burlingame, USA), used at 1:10,000; HRP-conjugated horse anti-rabbit IgG [H+L] (Vector Laboratories, Burlingame, USA), 1:10,000; and HRP-conjugated horse anti-goat IgG [H+L] (Vector Laboratories, Burlingame, USA), 1:5,000.

### High resolution respirometry

Oxygen consumption was measured using an O2K OROBOROS oxygraph (OROBOROS instruments, Innsbruck, Austria) as described in [17] with modifications. Briefly, whole fly homogenates were used for respirometry measurements. Briefly, 20–40 flies were homogenised in mitochondrial isolation buffer (250 mM sucrose, 5 mM Tris-HCl pH 7.4, 2 mM EGTA) and filtered before being immediately measured using an OROBOROS O2k oxygraph. Homogenates were incubated in assay buffer (120 mM KCl, 5 mM KH_2_PO_4_, 3 mM Hepes, 1 mM EGTA, 1 mM MgCl_2_, 0.2% bovine serum albumin, pH 7.2 at the same temperatures in which the flies were aged). State 4 respiration was measured by the addition of 5 mM pyruvate and 5 mM proline. State 3 was initiated with the addition of 1 mM ADP. CI-linked respiration was inhibited by 0.5 μM rotenone and 20 mM glycerol 3-phosphate was added to stimulate CIII-linked respiration. CIII-linked respiration was inhibited with the addition of 2.5 μM antimycin A. CIV respiration was initiated by the addition of 4 mM ascorbate and 2 mM TMPD. CIV respiration was inhibited with 0.5 mM KCN. Values were normalised to protein concentration as calculated by the Bradford method.

### Statistical analysis

**Fig 2:**
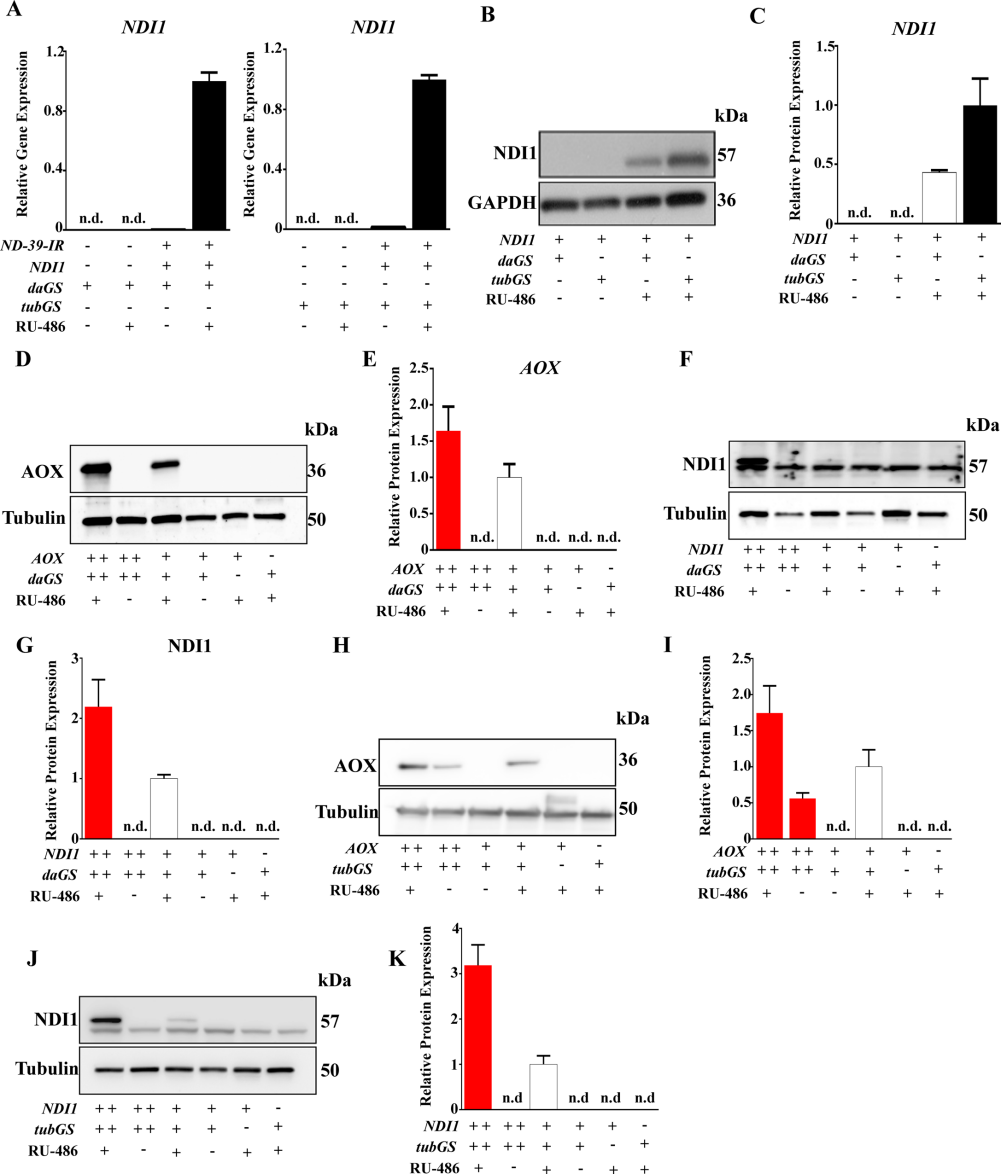
No significant leak is detected when expressing protein coding transgenes using GS. **(A**) qPCR expression data of NDI1 in induced vs. non-induced flies. Controls without the RNAi transgene are also included (n = 3). (B) Western blot analysis of NDI1 levels in induced vs non-induced flies. (C) Quantification of B (n = 2). (D) Western blot analysis of AOX expression driven by two (++) or one (+) copy of *daughterless*-GeneSwitch GAL4 (*daGS*) in flies carrying two (++) or one copy (+) of the AOX transgene in induced vs non-induced groups. (E) Quantification of D (n = 3). (F) Western blot analysis of NDI1 expression driven by two (++) or one (+) copy of *daughterless*-GeneSwitch GAL4 (*daGS*) in flies carrying two (++) or one copy (+) of the NDI1 gene in induced vs non-induced groups. (G) Quantification of F (n = 3). (H) Western blot analysis of AOX expression driven by two (++) or one (+) copy of *tubulin*-GeneSwitch GAL4 (*tubGS*) in flies carrying two (++) or one copy (+) of the AOX gene in induced vs non-induced groups. (I) Quantification of H (n = 3). (J) Western blot analysis of NDI1 expression driven by two (++) or one (+) copy of *tubulin*-GeneSwitch GAL4 (*tubGS*) in flies carrying two (++) or one copy (+) of the NDI1 gene in induced vs non-induced groups. (K) Quantification of J (n = 3). GAPDH or Tubulin is shown as loading control. +/- indicate presence/absence of the transgene or 500 μM RU-486 (inducer).

**Fig 3:**
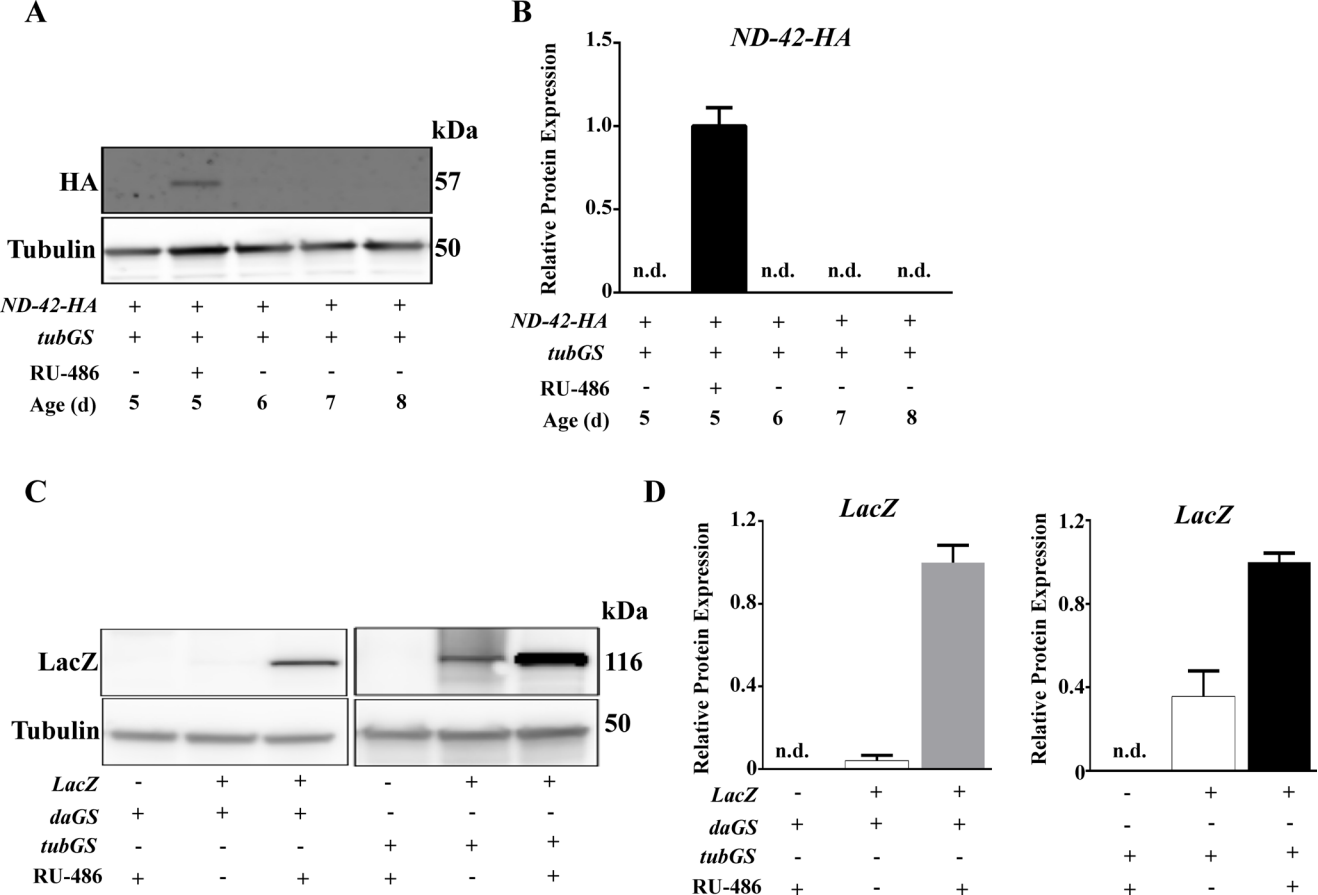
Expression of protein coding transgenes correlates with the presence of RU-486 in the fly food. (A) Western blot analysis of ND-42-HA levels after feeding flies RU-486 for 5 days and after withdrawal of the drug from the food. (B) Quantification of A (n = 3). (C) Western blot analysis of LacZ levels driven by *daGS* or *tubGS* GAL4 in induced vs non-induced groups. (D) Quantification of C (n = 3). Tubulin is shown as loading control. *daGS*, *daughterless*-GeneSwitch GAL4; *tubGS*, *tubulin*-GeneSwitch GAL4; +/- indicate presence/absence of the transgene or 500 μM RU-486 (inducer).

**Fig 4:**
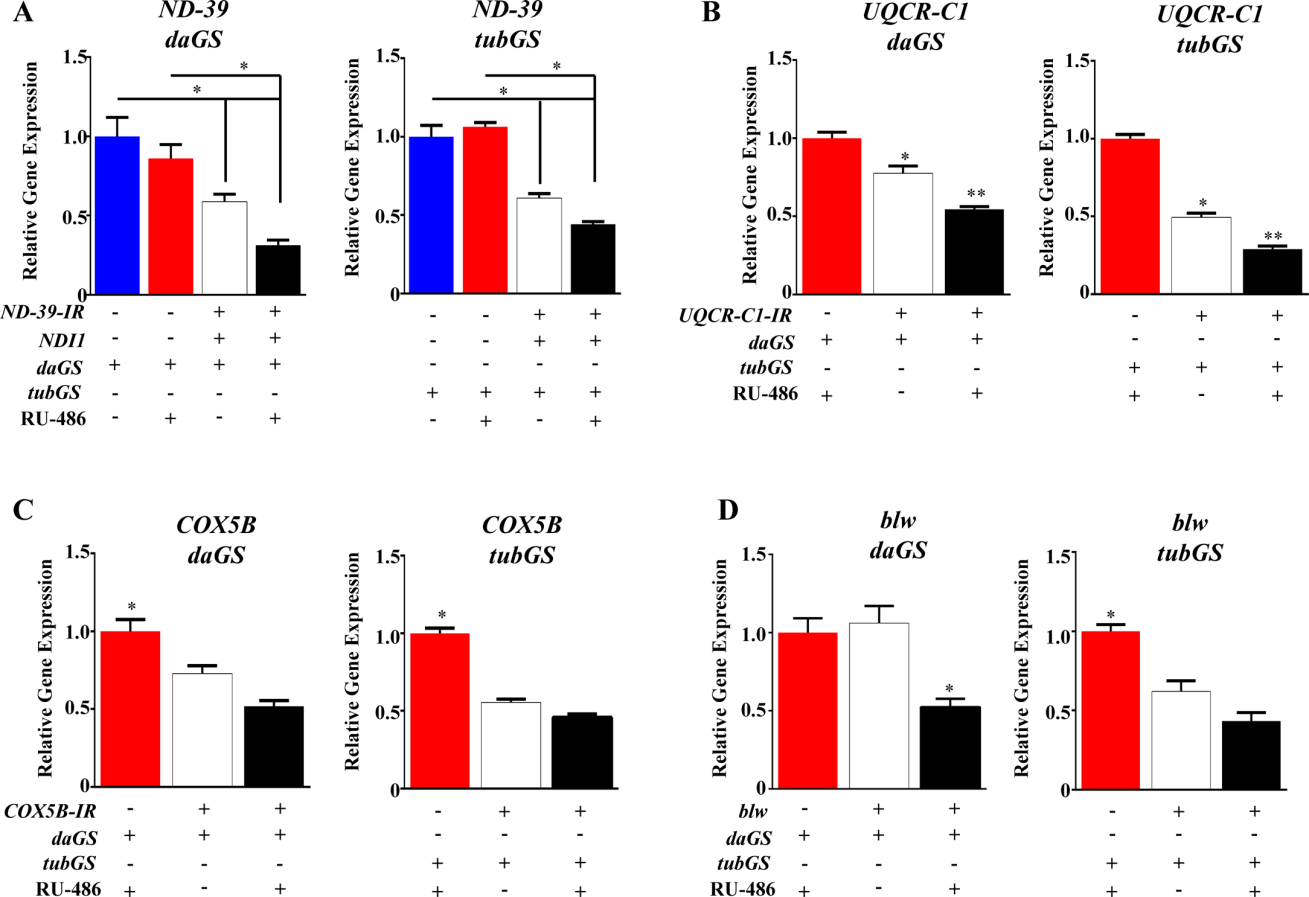
Use of qPCR to validate knock-down of a gene using GS requires additional controls. (A) qPCR expression data of *ND-39* (CI subunit) in induced vs non-induced groups (n = 3). (B) qPCR expression data of *UQCR-C1* (CIII subunit) in induced vs. non-induced groups (n = 3). (C) qPCR expression data of *COX5B* (CIV subunit) in induced vs. non-induced groups (n = 3). (D) qPCR expression data of *Bellwether* (CV subunit) in induced vs. non-induced groups (n = 3). *daGS*, *daughterless*-GeneSwitch GAL4; *tubGS*, *tubulin*-GeneSwitch GAL4; +/- indicate presence/absence of the transgene or 500 μM RU-486 (inducer).

**Fig 5:**
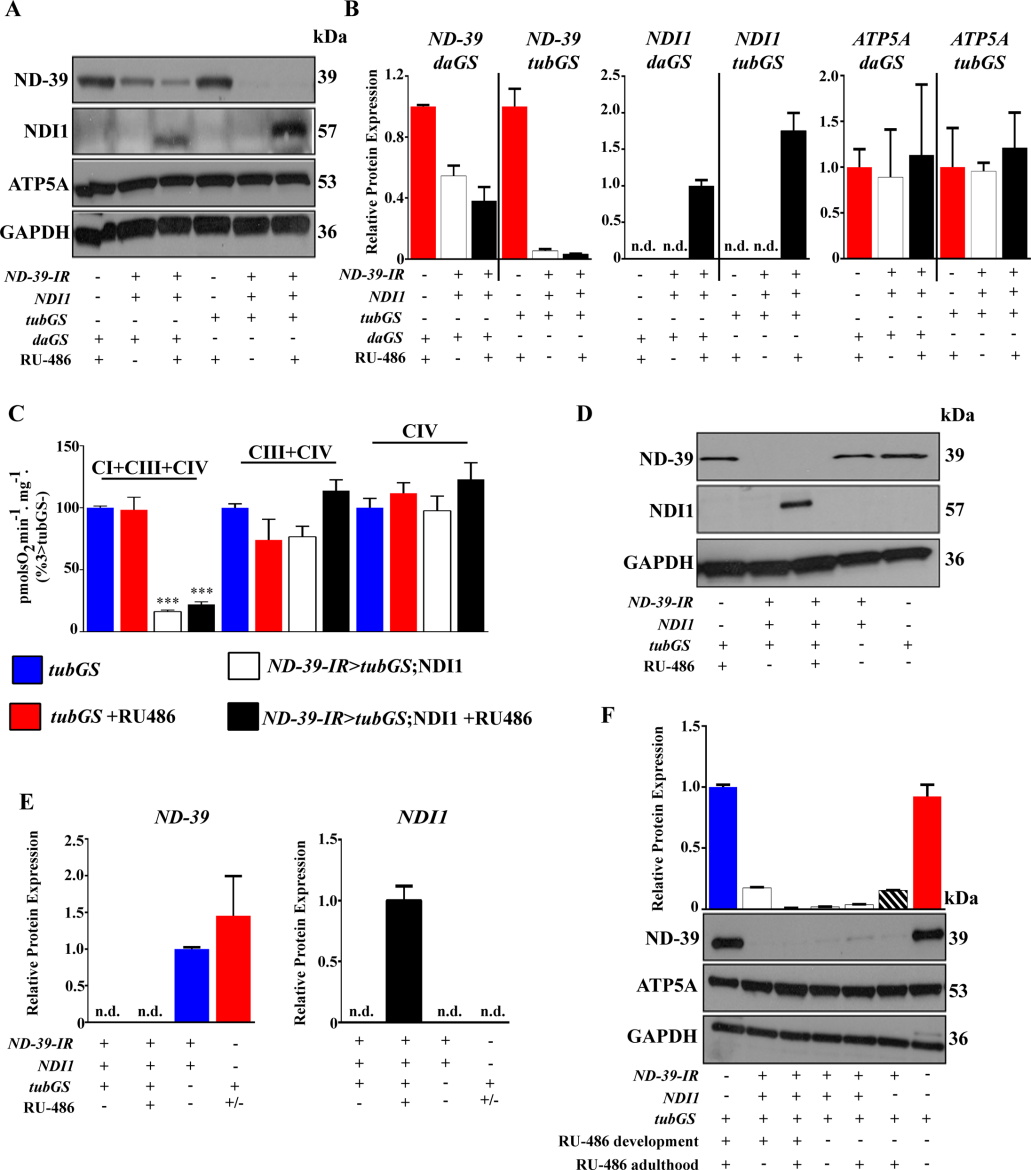
Expression of RNAi transgenes does not correlate with the presence of RU-486 in the fly food. (A) Western blot analysis of ND-39, NDI1 and ATP5A levels in control, induced and non-induced groups. (B) Quantification of A (n = 2). (C) Respirometry in *tubGS* flies with and without an ND-39 RNAi transgene (n = 3–8). (D) Western blot analysis of ND-39 and NDI1 levels in control, induced and non-induced groups. (E) Quantification of D (n = 2–3). (F) Western blot analysis of ND-39 levels in controls and experimental flies fed with RU-486 during development (1 μM), development and adulthood (1 μM and 500 μM respectively) or exclusively during adulthood (500 μM) (n = 2–3). All flies were 5 days old when proteins were extracted. Flies that were exclusively fed during development spent 5 days in food without RU-486. GAPDH is shown as loading control. *daGS*, *daughterless*-GeneSwitch GAL4; *tubGS*, *tubulin*-GeneSwitch GAL4; +/- indicate presence/absence of the transgene or 500 μM RU-486 (inducer).

**Fig 6:**
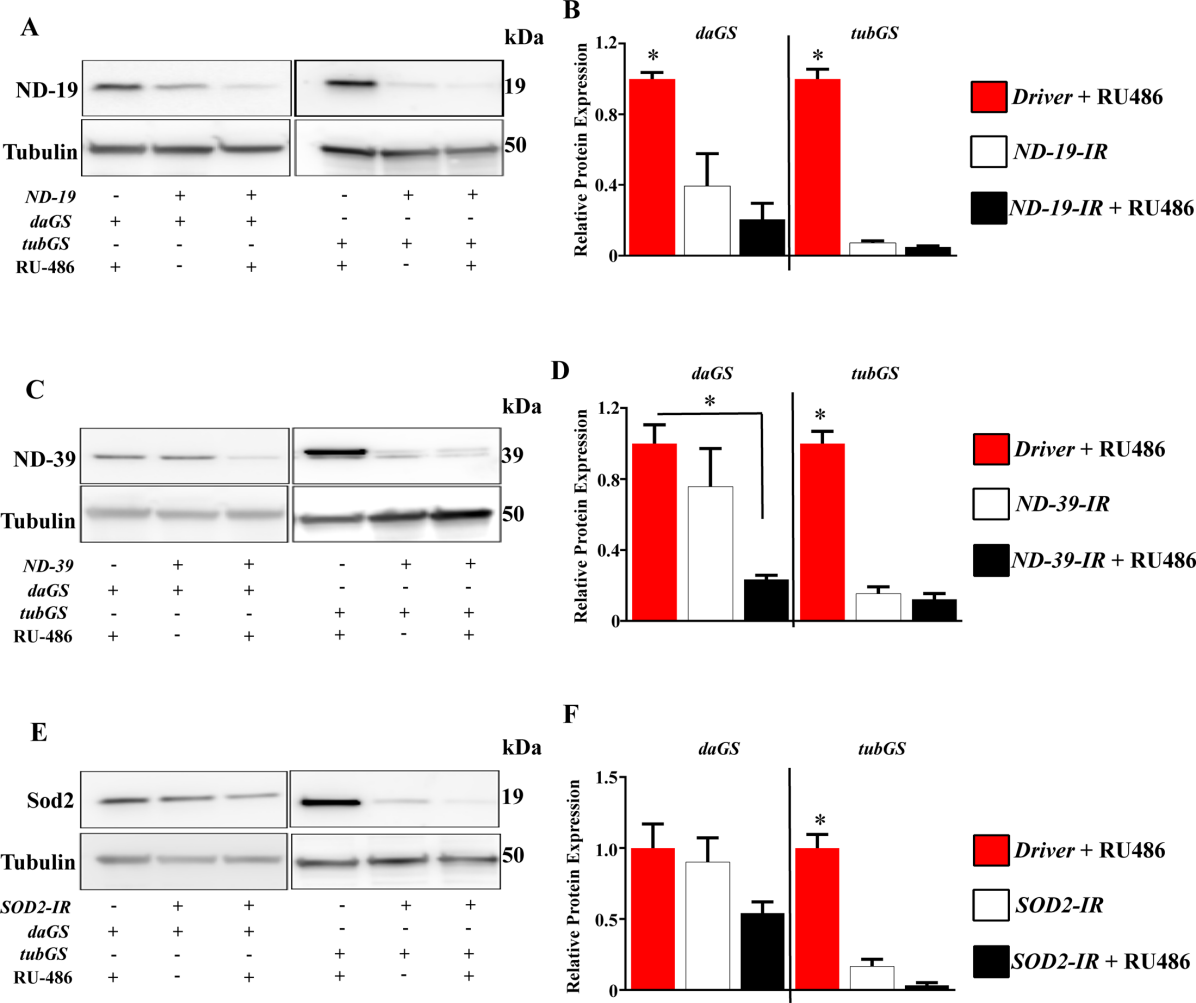
No difference in protein levels between induced and non-induced groups using a strong GS driver. (A) Western blot analysis of ND-19 levels in control (without the RNAi transgene), induced and non-induced groups. (B) Quantification of A (n = 3). (C) Western blot analysis of ND-39 levels in control (without the RNAi transgene), induced and non-induced groups. (D) Quantification of C (n = 3). (E) Western blot analysis of Sod2 levels in control (without the RNAi transgene), induced and non-induced groups. (D) Quantification of E (n = 3). Tubulin is shown as loading control. *daGS*, *daughterless*-GeneSwitch GAL4; *tubGS*, *tubulin*-GeneSwitch GAL4; +/- indicate presence/absence of the transgene or 500 μM RU-486 (inducer).

Data are shown as mean ± SEM. Data analysis was performed with Prism 6 (GraphPad) using either 1-way ANOVA with Newman-Keuls post-test or the unpaired Student’s *t*-test where appropriate. *p* values <0.05 were taken as statistically significant. A summary of the raw data for Figs 2–6 is shown as supplementary information in S1 Table. In all figures * = p<0.05 denotes significant difference from all other groups without * unless indicated otherwise by line art. n.d. = not detected.

## Results and Discussion

### The nature of the transgene dictates the extent of non-induced expression when using the GS system

GS has been used for the over-expression of endogenous and exogenous genes, with expression levels dependant on the activity of the promoter used to express the GAL4 transcription factor [8]. Accordingly, we used the *daughterless* (*daGS*) and the *tubulin* (*tubGS*) GS GAL4s to express the *alternative NADH dehydrogenase internal 1* (NDI1) from *Saccharomyces cerevisiae* [11] at low and high levels respectively. We did not observe significant expression in non-induced flies at the mRNA (Fig 2A) or protein level (Fig 2B and 2C). Even in the presence of two copies of the NDI1 transgene, no NDI1 was detected in absence of RU-486 (Fig 2F, 2G, 2J and 2K). Similar results were observed when we expressed another alternative respiratory enzyme -the alternative oxidase: AOX [16]- using *daGS* (Fig 2D and 2E). However, when *tubGS* was used we did observe expression non-induced in flies carrying two copies of the AOX transgene (Fig 2H and 2I). Next, using a transgene for ND-42 protein tagged with the human influenza hemagglutinin epitope (ND-42-HA) we were able to confirm that levels of gene expression correlated with the presence of RU-486 in the fly food and that expression of ND-42-HA was reversible upon withdrawal of RU-486 (Fig 3A and 3B). In the past, a detectable leak in expression was reported when exogenous genes such as *lacZ* or green fluorescence protein (GFP) were expressed using GS[9]. The extent of the leak was dependent on the Gal4 driver utilized. Unfortunately as neither *daGS* nor *tubGS* were used in this article[9] our results cannot be compared. Nevertheless, when a strong GS GAL4 driver such as *tubGS* was used we found significant expression of LacZ in non-induced conditions, (Fig 3C and 3D) recapitulating previously reported results [9].

Importantly, we observed a strikingly different response when RNAi transgenes, designed to inhibit mRNA translation, were expressed in the same experimental conditions. Using an RNAi against complex I subunit: *NADH dehydrogenase (ubiquinone) 39kDa subunit (ND-39*) as an example, we observed a significant depletion in protein levels independent of the presence of RU-486. Firstly, using quantitative real time PCR (q-RT-PCR), an approach commonly used to validate the efficiency of the knock-down using GS, we measured mRNA levels in induced (flies fed with RU-486) and non-induced flies [18, 19]. We found a clear decrease in the levels of expression of the target gene using both *daGS* and *tubGS* (Fig 4A). However, we found that this approach could be misleading as when compared with control flies which did not carry the RNAi transgene non-induced flies displayed a strong depletion of the target gene (Fig 4A). We also observed for a further three target genes, with the level of knock down dependant on the driver and RNAi construct used, with a stronger driver (i.e. *tubGS*) showing lower levels of the target gene (Fig 4B–4D). Analysis of protein levels by western blot revealed depletion of ND-39 in the absence of RU-486 (Fig 5A and 5B) mirroring results at the mRNA level. As expected, the level of target gene knock down was dependent on the strength of the driver used. In the case of the strong driver *tubGS*, the level of depletion was 95% to 97% for non-induced versus induced, whereas with the weaker driver *daGS* only 45% depletion was observed in non-induced flies (Fig 5B). Significantly, NDI1 was only present in induced flies (Fig 5A and 5B), demonstrating that the extent of non-induced expression depends on the nature of the construct. We further confirmed our results using high resolution respirometry[14]. Complex I (CI)-linked respiration was strongly reduced in flies carrying the RNAi transgene in combination with a GS driver independently of the presence of RU-486 (Fig 5C). The fact that only CI-linked respiration was affected demonstrates that this is a specific consequence of the presence of an RNAi transgene against a CI subunit, and not due to an unspecific effect of inducing an RNAi response. No effect on the protein levels of ND-39 (Fig 5D and 5E) or on CI-linked respiration (data not shown) was observed in flies carrying only the RNAi transgene without a GS driver, indicating that the combination of a GS driver with an RNAi transgene results in the effects we observed. In order to understand if it is possible to rescue normal expression of the target protein after induction of the RNAi expression, as we did for the tagged ND-42 protein shown in Fig 3A, we fed flies with RU-486 during development, development and adulthood or only during adulthood. We hypothesised that if the knock-down was reversible, removal of the drug after development would return protein levels to normal. However, we found that ND-39 was depleted to a similar level independently of when RU-486 was administrated, i.e. during development, during adulthood or in both stages (Fig 5F). This indicates that it is not possible to regulate protein levels using RNAi constructs in combination with GS, and that knock-down is not reversible using this system. We found this to be the case for a further two RNAi lines tested in the same conditions *ND-19 (CG3683)* and *Sod2* (*CG8905*) and for *ND-39* in the absence of NDI1 (Fig 6A–6F). For all three lines the decrease at the protein level was over 80% in absence of RU-486, when *tubGS* was used as driver. However, when *daGS* was used, the depletion in the non-induced flies was between 10 and 61%. A similar phenomenon has been reported for two RNAi lines *Prosβ5* (*CG12323*) and *Prosα7* (*CG1519*) which in combination with the *tubGS* driver caused developmental lethality even in absence of RU-486[20, 21]. Although surprising to the best of our knowledge no one has previously reported that the leak in the GS system is dependent on the nature of the transgene, so it is possible that the level of non-induced expression varies depending on the background and diet. We hypothesize that non-induced expression observed when RNAi transgenes are expressed may be due to a systemic amplification mechanism such as that which has been described in *Caenorhabditis elegans* [22]. In fruit flies however there is no clear evidence for the existence of either secondary small interference RNA or of the RNA-dependent RNA-polymerase (RdRP) that drives this process in worms (reviewed in [23]) and therefore the phenomenon we observe here remains unclear at the mechanistic level. The fact that many other laboratories including ourselves have reported phenotypic differences between induced and non-induced groups could be due to a variety of reasons. Firstly, we have used whole flies for our analysis so it is possible that some tissues or cell types are more refractory to the phenomenon we observe, and that the differences reported are due the variations in protein levels in these tissues. Secondly, it is also possible that the presence of inducer during development accelerates protein depletion and specific changes during this period are responsible for the phenotypes described. Indeed, differences in diet composition during development have strong effects on the body composition of adults [24]. Thirdly, it is also possible that there is an interaction between RU-486 and knock-down of specific genes. Finally, we have observed that high concentrations of RU-486 induce the generation of mitochondrial ROS (Scialo and Sanz, unpublished observation) that in interaction with the knock-down can explain the phenotypes previously reported.

### Final remarks

Dissecting the role of a gene requires manipulation of its function in different tissues and at different developmental stages which can be technically challenging. The GS system was generated to allow the induction of gene expression at specific time points during the fly life cycle and to solve issues related to genetic background. However, our studies indicate that the use of GS may present some technical challenges which prevent this when RNAi transgenes are expressed. Firstly, the selective leakiness of the system means that even when using weak GS Gal4 drivers (e.g. *daughterless*) the levels of the target protein are significantly reduced compared with control flies carrying only the RNAi transgene or the GS Gal4 driver. This necessitates additional controls negating the background advantage of using GS. However, the phenomenon described here does allow the study of the effects of overexpression of one or more genes in a background where other genes are being depleted. As an example, we demonstrate that it is possible to study the effect of ectopically expressed NDI1 in a background where ND-39 has been depleted. Similarly, other combinations of genes/proteins are possible, thus further increasing the experimental flexibility available when working with fruit flies. This would allow for genetic screens where overexpression of target genes is induced through the addition of RU-486 to the food, to rescue phenotypes resulting from RNAi depletion of target genes in the absence of RU-486. Using this approach it would be possible to test if knockdown of CI subunits can be rescued through for example the overexpression of mitophagy pathway components (e.g. Parkin, Pink1, Drp1, etc). Further combinations are possible, increasing the repertoire of tools available for manipulation of gene function in *Drosophila melanogaster*.

## Supporting Information

S1 Table Summary of raw data of Figs 2–6. (XLSX)
